# Holding All the Cards—How Fanconi Anemia Proteins Deal with Replication Stress and Preserve Genomic Stability

**DOI:** 10.3390/genes10020170

**Published:** 2019-02-22

**Authors:** Arindam Datta, Robert M. Brosh

**Affiliations:** Laboratory of Molecular Gerontology, National Institute on Aging, NIH, NIH Biomedical Research Center, Baltimore, MD 21224, USA; arindam.datta@nih.gov

**Keywords:** Fanconi anemia, genomic instability, DNA repair, DNA replication, genetic diseases, cancer, chromosome, helicase

## Abstract

Fanconi anemia (FA) is a hereditary chromosomal instability disorder often displaying congenital abnormalities and characterized by a predisposition to progressive bone marrow failure (BMF) and cancer. Over the last 25 years since the discovery of the first linkage of genetic mutations to FA, its molecular genetic landscape has expanded tremendously as it became apparent that FA is a disease characterized by a defect in a specific DNA repair pathway responsible for the correction of covalent cross-links between the two complementary strands of the DNA double helix. This pathway has become increasingly complex, with the discovery of now over 20 FA-linked genes implicated in interstrand cross-link (ICL) repair. Moreover, gene products known to be involved in double-strand break (DSB) repair, mismatch repair (MMR), and nucleotide excision repair (NER) play roles in the ICL response and repair of associated DNA damage. While ICL repair is predominantly coupled with DNA replication, it also can occur in non-replicating cells. DNA damage accumulation and hematopoietic stem cell failure are thought to contribute to the increased inflammation and oxidative stress prevalent in FA. Adding to its confounding nature, certain FA gene products are also engaged in the response to replication stress, caused endogenously or by agents other than ICL-inducing drugs. In this review, we discuss the mechanistic aspects of the FA pathway and the molecular defects leading to elevated replication stress believed to underlie the cellular phenotypes and clinical features of FA.

## 1. FA Pathway and Models of Interstrand Cross-link Repair

Fanconi anemia (FA) is a hereditary disorder characterized by congenital abnormalities, progressive bone marrow failure (BMF), and predisposition to cancer, especially leukemia and aggressive head and neck squamous cell carcinoma (HNSCC) [1,2,3]. Head and neck squamous cell carcinoma is thought to arise within a geographical field of the mucosal epithelium, leading to tumors with chromosomal instability due to a deficiency in the FA pathway; contributions of human papilloma virus infection as well as genotoxic stress may contribute, but this is debated [3] Leukemia is thought to arise from chromosomal aberrations in the hematopoietic stem cells, leading to leukemic transformation by the rise of clonal populations [4] More research in this area is required to address the predominant origins of cancers observed in FA. Fanconi anemia patient-derived cells are extremely hypersensitive to agents that generate DNA interstrand cross-links (ICLs) that covalently bond the two strands of the DNA double helix to one another [5,6,7,8,9] There is a spectrum of structurally distorting and non-distorting DNA ICLs caused by endogenous sources that differ in their target sequences [10] Therefore, it is plausible that the physical structure at the ICL mediates the proteins that are involved in ICL unhooking and repair (discussed below). In addition, a variety of DNA lesions including ICLs can be induced by exogenous chemicals including cancer chemotherapy drugs (nitrogen mustards, cisplatin, mitomycin C (MMC), psoralen) that also differ in their target sequences and site-specific adducts [10] Among the agents leading to covalently cross-linked DNA and protein-DNA adducts is formaldehyde, which can arise endogenously. For a perspective on the chromosomal instability and hematopoietic stem cell attrition caused by formaldehyde, readers are referred to several recently published papers from the Patel lab and the references therein [11,12]. Emerging evidence suggests that the clinical and cellular features of FA are caused not only directly by ICL-induced chromosomal instability but also more globally by inflammation and defects in epigenetic regulation and telomere metabolism [13]. 

The connection of FA to accumulation of ICLs has prompted much interest in the source of ICLs and mechanism(s) for repair. Research in simple unicellular organisms such as yeast and bacteria has provided insight. Studies of *E. coli* dating back to the 1970s led researchers to believe that ICLs are repaired by both nucleotide excision repair (NER) and homologous recombination (HR) in sequential steps, but the details were unclear [14,15,16]. Later, it was proposed that ICL repair could also occur in nonreplicating bacteria by a non-recombinogenic mechanism requiring a translesion (TLS) DNA polymerase [17,18]. Moving to simple eukaryotes, research from a number of laboratories suggests that ICL repair in yeast is likely to be more complex, with a greater number of proteins from a more expansive list of classical repair pathways involved [19,20]. 

**Figure 1:**
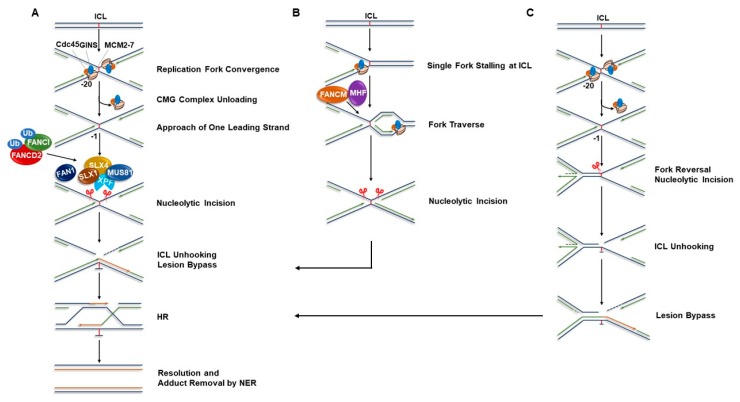
Models of replication-coupled ICL repair. In replicating cells, ICLs are efficiently repaired by cooperative action of FA pathway proteins and the proteins involved in the NER, TLS, and HR pathways. Other than the classical ICL repair model of single fork convergence, there are different proposed mechanisms by which crosslinks can be repaired in a replication-dependent manner, enabling forks stalled at ICLs to resume DNA synthesis. (**A**) Dual fork convergence model. Crosslink repair is triggered when two replication forks converge at an ICL. The leading strands of two converging forks initially stall at ~20 nucleotides away from the lesion due to the steric hindrance imposed by the CMG complex (Cdc45/MCM2-7/GINS). Ubiquitin signaling promotes chromatin unloading of the CMG complex, which allows one of the leading strands on either side of the ICL to approach to within one nucleotide of the lesion. The FA pathway is simultaneously activated through the monoubiquitination of the FANCI‒FANCD2 complex. Ubiquitylated FANCI‒FANCD2 in turn promotes recruitment of SLX4 (FANCP), which acts as a molecular scaffold to recruit structure-specific endonucleases SLX1, XPF (FANCQ)-ERCC1, and MUS81 at the ICL site. Nucleolytic incision and subsequent unhooking of the ICL by the coordinated actions of these enzymes creates a DSB in one sister chromatid. FAN1, another endonuclease, is also implicated in the incision step. TLS polymerases, Pol ζ and REV1 subsequently extend the leading strand past the unhooked ICL and generate an intact DNA template suitable for HR repair. The DSB is finally repaired by HR and the unhooked ICL is removed by NER process. (**B**) Replication traverse model. In living cells, the majority (~60%) of replication forks encountering ICLs bypass the lesions without unhooking them in a manner dependent on the translocase activity of FANCM/MHF complex. The “X-shaped” DNA structures generated through the traverse process are subsequently processed by endonucleases followed by lesion bypass and post-replication repair as in dual fork convergence model. (**C**) Replication fork reversal at ICL. Following CMG complex unloading, one of the two converging forks undergoes reversal. The opposite fork is subsequently incised by the endonucleases leading to ICL unhooking. The lesion is then bypassed by the action of TLS polymerases and DSB intermediate generated during the incision process is finally repaired by HR. ICL: Interstrand cross-link; FA: Fanconi anemia; NER: Nucleotide excision repair; TLS: Translesion; HR: Homologous recombination.

Since the discovery of the first FA gene *FANCC* over 25 years ago [21], mutations in a growing list of genes (*FANCA*, *FANCB*, *FANCC*, *FANCD1*, *FANCD2*, *FANCE*, *FANCF*, *FANCG*, *FANCI*, *FANCJ*, *FANCL*, *FANCN*, *FANCP*, *FANCQ*, and *FANCT*) are linked to FA. In addition, mutations in three genes (*FANCO*, *RAD51* (*FANCR*), and *FANCS*) are implicated in a FA-like syndrome in which patient cells are hypersensitive to ICL-inducing drugs, but the disease does not present with all the clinical features classically observed in FA [22,23]. Collectively, the FA gene products comprise a specialized pathway of ICL repair (Figure 1). In addition to these genes, *FANCM* is involved in ICL repair with other proteins in the FA pathway; however, it is considered an atypical FA gene because its linkage to FA has not been formally demonstrated [8,21]. Among the bona fide FA genes, *FANCA*, *FANCC*, and *FANCG* are most frequently inactivated by bi-allelic mutations linked to the hereditary disorder (Fanconi Anemia Database; http://www2.rockefeller.edu/fanconi/) [24]. Importantly, mono-allelic mutations in certain FA genes including *FANCD1* (*BRCA2*)*, FANCS* (*BRCA1*), *FANCN* (*PALB2*), *FANCM*, *FANCJ* (*BRIP1*), and *FANCO* (*RAD51C*) that are believed to operate downstream in the pathway and implicated in HR are associated with sporadic breast and ovarian cancer [24,25].

Proteins implicated in the FA pathway primarily operate during the DNA synthesis (S)-phase of the cell cycle and cooperate with structure-specific endonucleases, DNA polymerases implicated in TLS, and HR proteins to fix the double-strand breaks (DSBs) that arise from processed ICLs. Although the dominant pathway of ICL repair in proliferating cells is replication-dependent, nondividing or slowly diving cells such as neurons and stem cells may employ a pathway of replication-independent repair (RIR) to correct these toxic lesions [26,27,28]. RIR operates through the coordination of TLS and NER, and requires DNA polymerase κ (Pol κ), DNA polymerase ζ (Pol ζ), and an ATP-dependent DNA translocase known as Cockayne syndrome Group B protein (CSB) to repair ICLs outside of S-phase [27,28,29]. The precise roles of FA gene products usually associated with the classical replication-dependent pathway in RIR remain to be thoroughly understood.

In the classical FA pathway of ICL repair, ICL-blocked replication forks are first recognized by a FANCM‒FAAP24‒MHF1‒MHF2 protein complex, which in turn activates the FA core complex to mono-ubiquitinate FANCD2-I heterodimer (ID-Ub); this represents a key signaling step in the FA pathway [8,30,31]. FANCD2-I mono-ubiquitination is followed by a series of events including ID-Ub-dependent incision and unhooking of the ICL by endonucleases, followed by TLS, and HR-mediated repair of the DSB intermediates. However, several models for processing of an ICL-stalled X-shaped replication fork have been proposed and these are being actively investigated (Figure 1). The simplest model depicts a single replication fork encounter of an ICL followed by unhooking, TLS, and fork restoration via HR [32,33]. Importantly, this model first delineated roles of FANCM (a DNA translocase) and FANCJ (a bona fide DNA helicase) in the FA pathway of ICL repair. FANCM was speculated to facilitate endonucleolytic cleavage by disrupting duplex DNA surrounding the ICL, whereas FANCJ was proposed to operate with other HR proteins to rejoin DSBs by a homologous DNA sequence-dependent pathway. However, this classical ICL-repair model lacked some mechanistic details regarding the precise roles of FA proteins and replisome proteins in the repair process. 

More recently, a second model of ICL repair was proposed. Raschel et al. performed reconstitution experiments with *Xenopus* egg extract that was incubated with a plasmid carrying a single, site-specific ICL; they demonstrated that repair is triggered when two replication forks collide with the ICL [9]. In this dual fork convergence model of ICL repair (Figure 1A), the leading strands of two converging replication forks are initially stalled at ~20‒40 nucleotides (“-20 position”) away from the lesion due to steric hindrance imposed by the template bound CMG (CDC45, MCM2-7, and GINS) replicative helicase. Subsequent eviction of the CMG complex from the DNA allows the leading strands to approach further and extend up to one nucleotide away from the ICL (“-1 position”). The HR protein BRCA1 has been proposed to play a crucial role at this step by promoting unloading of the CMG complex, thereby paving the way for leading strand synthesis to extend towards the ICL [34]. Concurrent activation of the FA pathway via mono-ubiquitylation of the FANCD2-I complex in turn promotes the incision of one of the parental strands by XPF‒ERCC1 and another incision in the same strand by possibly another endonuclease(s), thereby “unhooking” the ICL and creating a DSB. TLS polymerases such as DNA polymerase ζ and REV1 facilitate lesion bypass on the opposite strand and recreate an intact duplex that serves as a template for subsequent HR-mediated repair of the DSB. The DSB is finally repaired by HR and the unhooked ICL remnant is removed by NER. This model proposes that the X-shaped structure formed when two replication forks converge at an ICL is the essential triggering factor for the repair process to begin. This mechanism was further supported by a subsequent study where a single replication fork stalled at an ICL was shown to be unable to promote ICL repair in a cell-free *Xenopus* egg extract [35]. Further studies are required to tease out the precise mechanistic details of the dual convergence model that operate in vivo. Moreover, it remains to be determined how precisely conserved are the molecular events of ICL repair in the *Xenopus* reconstituted system compared to mammalian cells.

Given the uncoupling of the CMG replicative helicase prior to ICL repair, the question has been raised how replication resumes without CMG loading following fork recovery unless another fork from the opposite direction arrives. This points towards the logical validity of the dual-fork convergence concept. However, given the long inter-origin distance (~100 kb) in eukaryotic cells, simultaneous arrival of two replication forks at an ICL seems unlikely and it is conceivable that one fork encounters the barrier before another fork arrives in vivo [36]. Therefore, both single fork collision and dual-fork convergence at ICLs are possible, but the relative frequencies might vary. Importantly, productive ICL repair was shown to occur in *Xenopus* egg extracts even when there was a significant time gap between the arrival of the two forks at an ICL [35]. In 2013, Seidman and colleagues proposed a new concept of ICL repair, which they termed “replication traverse” [37]. Using a specialized DNA fiber technique, they monitored DNA replication tracts at fluorescently labeled ICLs in living cells and showed that instead of ICL “unhooking”, the majority of the replication forks (~60%) bypass or “traverse” the ICLs in a manner dependent on the translocase activity of FANCM/MHF complex (Figure 1B). The remnant ICLs are likely to be removed through post-replication repair. ICL traverse of the replisome is essentially dependent on the functional interaction between FANCM and the Bloom’s syndrome helicase complex (BLM, Topoisomerase 3α, RMI1 and RMI2); together, the proteins operate in an epistatic manner in crosslink repair [38]. In a parallel study, Kisker’s group further showed that the replication traverse is dependent on the functional interaction of FANCM with the replicative polymerase co-factor PCNA [39]. Interestingly, FANCM plays apparently distinct roles in replication-transverse and the classical FA pathway of ICL repair. In addition to FANCM’s role as a DNA translocase to promote replication-traverse, FANCM also facilitates chromatin recruitment of the FA core complex and subsequent FANCD2-I mono-ubiquitination in response to ICL-induced DNA damage [40,41,42]. However, cells deficient of FANCM are less sensitive to ICLs compared to cells with an inactivated FA core complex, suggesting that replication traverse plays a less protective role than the conventional FA pathway when the replication machinery encounters an ICL [40,43].

Although conventional wisdom would argue that the replisome machinery cannot continue to synthesize DNA on the other side of a DNA ICL, experimental data supporting the Seidman model for replication traverse have recently been generated by the Lopes lab and provide further mechanistic insight [44]. Fork traverse requires global fork slowing mediated by fork reversal enacted by the double-stranded DNA translocase ZRANB3 and HR protein RAD51 as well as ATR checkpoint activation. Moreover, as reported by the Seidman group [37], FANCM was shown to promote efficient fork traverse thereby acting as a stimulatory component of the specialized fork restart pathway. Determination of the precise ATR targets supporting replisome progression and stability remains to be seen.

The molecular mechanism(s) of ICL repair are still being investigated and a direct comparison of the processing efficiency of single fork, dual fork, and traversed fork at ICLs would provide insight. Using Xenopus egg extracts, the Walter group showed that a classic psoralen-induced ICL or an abasic site ICL characterized by a nonnative *N*-glycosyl bond can be “unhooked” by NEIL3, a DNA glycosylase [45]. This distinct mechanism of replication coupled-ICL repair does not require FANCI-FANCD2-dependent incision, thereby preventing generation of DSB intermediates. In another interesting study, they further demonstrated that one of the two converging forks at an ICL undergoes reversal in a manner that is dependent on prior removal of the CMG from the template strand (Figure 1C) [46]. In this context, Lopes and co-workers recently reported that ICL induction causes replication fork slowing and reversal at crosslink sites as well as non-ICL sites [44]. Using DIG-TMP (Digoxygenin-tagged TMP)-based ICL immunolabeling combined with DNA fiber assay and electron microscopy, the authors provided experimental evidence that in response to ICLs, ATR-mediated global fork slowing rapidly recruits RAD51 to chromatin leading to fork reversal. This in turn facilitates single-fork ICL traverse and prevents chromosomal instability. This new information not only updated the existing dual fork convergence model of ICL repair but also presented a new avenue of research, namely fork protection in the context of ICL repair. Certain FA proteins (BRCA2, RAD51, FANCD2) and associated factors (DNA helicase-nuclease proteins DNA2 and WRN (the latter mutated in the premature aging disorder Werner syndrome [47]) protect forks from dysregulated degradation, which is critically important for genomic stability [48,49,50,51,52,53]. Therefore, it will be important to delineate the functions of WRN and DNA2 in fork protection at sites of ICLs.

## 2. Functional Involvement of FA Proteins with Players in Other DNA Repair Pathways

Functional integration of FA proteins with NER, TLS, and HR repair factors is indispensable for ICL repair. In addition, many proteins of the FA pathway collaborate with these repair factors to maintain genome integrity in response to non-ICL directed DNA damage. 

### 2.1. Nucleotide Excision Repair

In eukaryotes, helix-distorting bulky DNA lesions induced by UV exposure or platinum-based cancer chemotherapeutics are primarily repaired by the NER pathway [54,55]. Bi-allelic mutations in NER genes cause Xeroderma pigmentosum (XP), an autosomal recessive disorder characterized by impaired tolerance to UV light and predisposition to skin cancer [56]. XP patient-derived cells are hypersensitive to ICL-generating drugs, suggesting a functional role of NER proteins in DNA cross-link repair [29,57]. Consistent with this, cells inactivated of structure-specific NER endonucleases XPF‒ERCC1 or MUS81‒EME1 are hypersensitive to DNA crosslinking agents [57,58,59,60,61]. Moreover, *ERCC1*-deficient mice are prone to develop a hematopoietic defect like that observed in FA, implicating a direct role of the heterodimeric XPF‒ERCC1 endonuclease complex in the FA pathway [62]. Importantly, FA patients with mutations in the *ERCC4 (FANCQ)* gene encoding XPF endonuclease have been reported [63] and *ERCC4* restoration in FA patient-derived cells has been shown to complement MMC sensitivity [63,64]. At the mechanistic level, the ERCC1‒XPF nuclease complex acts in concert with FANCD2 and SLX4/FANCP to incise ICLs in the FA pathway [8,64,65,66]. Ubiquitylated FANCD2 facilitates the recruitment and activation of structure-specific nucleases including XPF‒ERCC1 and MUS81‒EME1 via the SLX4 scaffolding protein at the site of the crosslink and subsequently promotes ICL “unhooking,” thereby generating DSB intermediates. Consistent with the role of MUS81‒EME1 in the incision step, DSB induction is severely compromised in *MUS81* -/- mouse embryonic stem (ES) cells upon MMC or cisplatin treatment [58]. Moreover, the in vitro purified MUS81‒EME1 complex has been shown to cause an incision at the 5´ side of psoralen-induced ICL [67]. Experimental evidence, however, also suggests involvement of the structure-specific endonucleases in the downstream HR-mediated repair of the ICL-induced DSB intermediates. In agreement with this notion, *ERCC1-/*- mouse embryonic fibroblast cells (MEFs) display accumulation of DSBs upon ICL induction [61]. Furthermore, cells depleted of SLX4 or SLX1 are defective in resolving cisplatin-induced DSBs, implying the role of these nucleases in the downstream HR repair step. Likewise, experimental evidence suggests molecular links between Mus81 and HR-mediated repair as MUS81 has been shown to physically interact with the HR factor RAD54 [58,68]. Overall, these pieces of evidence suggest that the observed ICL hypersensitivity of human cells lacking structure-specific endonucleases is attributed not only to the defects in the initial incision step but also to the inefficient downstream processing of the incision-induced DSB intermediates.

Certain FA proteins facilitate NER of bulky DNA adducts such as UV photoproducts. BRCA1 (FANCS)-deficient cells are hypersensitive to UV-C-induced damage, suggesting its role in photoproduct elimination [69]. BRCA1 promotes XPF‒ERCC1 recruitment to replication forks stalled at unrepaired UV lesions and facilitates lesion excision in a replication-dependent manner [54]. Two additional FA components, the ubiquitin-conjugating enzyme UBE2T and the DNA translocase FANCM, also influence NER [70]. Genetic dissection experiments in avian DT-40 cells revealed that these two FA proteins cooperatively promote NER of UV-induced DNA damage and are important for protecting cells from UV genotoxicity. However, *FANCM*-deficient chicken DT-40 cells were found to be as effective as wild-type cells in photolesion removal. This suggests that FANCM likely functions downstream of the lesion excision step in the NER repair pathway. In contrast, UBE2T possibly facilitates UV lesion excision as *UBE2T* null cells were found to be defective in lesion removal, but the mechanistic role of the ubiquitin-conjugating enzyme to confer UV resistance is still poorly understood. Nonetheless, the collective observations suggest that the interaction of protein players in the FA and NER pathways plays a pivotal role in repairing DNA cross-links and other bulky DNA lesions. 

### 2.2. Double-Strand Break Repair

The functional link between FA and HR proteins in a common ICL repair pathway first became evident when FA patients with biallelic mutations in *BRCA2*, a key player in HR-mediated DSB repair, were reported [71]. This finding assigned *BRCA2* as a bona fide FA gene, designated *FANCD1*. Subsequent clinical and mouse genetic studies revealed that *BRCA2* loss gives rise to FA-like phenotypes including chromosomal abnormalities and robust hematological defects [72,73,74]. Genotype‒phenotype correlation studies in FA or FA-like patients further demonstrated functional roles for other HR factors including BRCA1 (FANCS), PALB2 (FANCN), RAD51 (FANCR), and FANCJ in ICL repair pathway [51,75,76,77,78,79,80,81,82,83]. BRCA1 in conjunction with PALB2, BRCA2, and RAD51 mediates the necessary downstream HR-dependent DSB repair events in the FA pathway [10,84,85,86,87]. However, BRCA1 has additional HR-independent functions upstream of the FANCD2-I monoubiquitylation event. Cell-based experiments provided evidence that BRCA1 is important for FANCD2 recruitment to ICL sites [88,89,90]. Moreover, BRCA1 promotes CMG helicase eviction from an ICL-stalled fork, an early step necessary for the repair process to begin [34]. FANCJ, like BRCA1, may also play an early role in the ICL response to facilitate chromatin loading of monoubiquitinated FANCD2 [91]. Zhang et al. presented data suggesting that cells expressing a FANCJ mutant specifically defective in its interaction with BRCA1 displayed impaired FANCD2 nuclear foci formation in unperturbed conditions as well as upon MMC treatment [92], consistent with coordinate involvement of FANCJ and its interacting partner BRCA1.

As opposed to its upstream role in the activation of the FA pathway, FANCD2 is also implicated in regulating HR-mediated repair of DSB intermediates formed during ICL repair, where it facilitates DNA end-resection through direct interactions with the CtIP and DNA2 nucleases [93,94,95]. In addition to its role in DSB processing, CtIP is involved in the initiation events of ICL repair in a BRCA1- and FANCM-dependent manner [89]. Thus, evidence suggests that HR proteins function both upstream and downstream of the FA core complex in the classical FA pathway of ICL repair. As the model of ICL repair is evolving, further research will provide more significant insights into the multi-level functional crosstalk between HR and FA proteins in repairing DNA crosslinks. Importantly, FA pathway components also contribute to repairing ICL-independent DSBs. FA proteins believed to operate both upstream (FANCA and FANCG) and downstream (FANCD2) in the pathway have been shown to promote HR-mediated repair of DSBs [96,97]. However, unlike *BRCA1*, *BRCA2,* and *RAD51*, mutations in the FA core complex or FANCD2 monoubiquitylation sites do not result in a severe HR defect, suggesting a non-essential role of upstream FA components in HR repair [97,98,99,100,101]. 

Although autosomal recessive mutations in certain RecQ helicase genes (*WRN*, *BLM*, *RECQL4*) are linked to accelerated aging diseases characterized by cancer predisposition, chromosomal instability, and a heightened sensitivity to DNA cross-linking agents and other genotoxic drugs, they are not genetically implicated in FA [102]. WRN, like BLM and RECQL4, plays functional roles in DSB repair that are likely to influence the downstream events of ICL repair [103,104]. Given its role in DNA end-resection in conjunction with DNA2, WRN might play an important role in generating 3´ ssDNA overhangs at DSB sites to initiate HR repair of the DSB intermediates [105]. On the other hand, WRN might also help in resolving HR products generated during the repair of DSB intermediates, as suggested by studies from the Monnat lab [104]. Indeed, WRN collaborates with BRCA1 in ICL repair [106], and it is conceivable that WRN might play a backup role to the FA pathway. In support of this idea, our laboratory showed that pharmacological suppression of WRN-catalyzed DNA unwinding by a WRN-specific helicase inhibitor (WRNi) compromises the cellular ICL response when the FA pathway is defective as evidenced by the hypersensitivity of *FANCD2* or *FANCA* mutant cells to MMC upon WRNi treatment [107]. It is conceivable that WRN helps to resolve HR intermediates formed at the later stage of ICL repair and loss of WRN’s helicase function impairs homology-directed ICL repair leading to activation of nonhomologous end-joining (NHEJ) repair, particularly when a protein of the FA pathway (e.g., FANCD2, FANCA) is defective. 

Aside from promoting HR-mediated DSB repair, the FA pathway likely suppresses the error-prone NHEJ mode of DNA repair [108,109,110]. In addition, data obtained from cell-based reporter assays suggest an important role of FA pathway components to promote alternative end-joining pathway of DSB repair [110]. In this context, Benitez et al. reported that FANCA plays a direct role in DSB repair by catalyzing single-strand annealing (SSA) and strand exchange (SE) in a FANCG-dependent manner [111]. Consistent with this, patient-derived FANCA mutants were found to be defective in both SSA and SE activities. Collectively, these observations suggest that FA pathway proteins ensure genome stability by promoting error-free pathways of DSB repair. 

### 2.3. Mismatch Repair

Functional interplay between the FA and mismatch repair (MMR) pathways is important for a robust response to agents that induce ICLs. Components of MMR protein complexes, specifically MutSα and MutSβ, are involved in ICL recognition as well as unhooking and HR-mediated repair of the processed ICL-induced DSBs [112,113,114,115]. Furthermore, physical interactions of MMR proteins with FANCD2, BRCA1, FANCJ, and SLX4 (FANCP) have been reported [116,117,118,119,120,121,122]. Along with other DNA repair proteins, MMR factors are integral components of the BRCA1-associated genome surveillance complex (BASC), which is believed to act as a crucial sensor for cellular DNA damage [116]. Mass spectrometry analyses of BRCA1 immunoprecipitated proteins from HeLa nuclear extracts revealed physical interactions between BRCA1 and the MMR proteins MSH2, MSH6, and MLH1 [116]. As a part of the BASC complex, the MSH2‒MSH6 heterodimer may recognize ICL-induced distorted helix structures and trigger DNA damage response signaling. Perhaps BRCA1 regulates DNA damage sensing by the MMR proteins or conversely these MMR factors might help BRCA1 to execute its repair functions in the FA pathway. High-throughput proteomic studies provided evidence for molecular interactions of MMR proteins with the structure-specific endonuclease FAN1 and scaffolding protein SLX4 implicated in FA [99,117,123,124]. LC-MS/MS-based proteomic analyses of HA-tagged SLX4 immune complex isolated from 293TREX cells showed that MSH2 and MSH3 reside in a large multimeric protein complex formed by SLX4 [117]. Moreover, protein‒protein interaction studies based on a tandem affinity purification (TAP) technique demonstrated that FAN1 interacts with MLH1 and PMS2 [123,124].

FANCJ directly interacts with MLH1, a component of the MutLα complex, through its helicase domain [122]. This interaction is essential for ICL repair as an impaired FANCJ‒MLH1 interaction renders cells sensitive to DNA cross-linking agents [122]. The MMR protein MSH2 is important in activating the FA pathway following ICL generation [121]. Although interactions of MSH2 and MLH1 with FANCD2 are both induced by DNA crosslinks, only MSH2 is important for FA pathway activation [121]. These findings suggest that while MSH2 functions upstream of FANCD2 monoubiquitination and triggers FA pathway activation, MLH1 functions downstream in the FA pathway and facilitates the repair of DSB intermediates. Consistent with this, either MSH2-deficient or MLH1-deficient cells are sensitive to ICL-inducing agents. Interestingly, MSH2 depletion in cells lacking a functional FANCJ‒MLH1 interaction suppresses ICL sensitivity and promotes replication restart [125]. Therefore, the MSH2-dependent toxic DNA damage response renders FA-defective cells sensitive to DNA cross-linking agents. Thus, an accurate functional coordination between FA and MMR pathways is critical to suppress a deleterious DNA damage response to ICLs. 

## 3. Involvement of the FA Pathway in Replication Fork Stabilization

When replication forks encounter DNA damage, an unusual DNA structure (e.g., G-quadruplex), or a transcribing RNA polymerase complex, the fork can be hindered in its progression. Stalled forks can also arise when cells are exposed to a DNA polymerase inhibitor (e.g., aphidicolin (APH)), an agent that causes nucleotide pool depletion (e.g., HU), or oncogene activation. Different structures form at stalled replication forks, depending on the nature of the block. In these situations, certain proteins are recruited to the site of the stalled fork to help manage the cellular response to the distress. Perhaps the myriad of DNA structures (e.g., leading strand gap, lagging strand gap, non-canonical DNA structure stabilized by Hoogsteen hydrogen bonds, regressed four-stranded Holliday Junction-like structure, etc.) helps to explain the diversity and redundancy of factors involved in replication fork protection. The metabolism of the stalled fork is growing to be much more complex than previously thought, but in simplest terms, a stalled fork center is dealt with by proteins and events that are focused on preserving the fork’s integrity while enforcing appropriate cell cycle checkpoints and allowing DNA synthesis to resume as efficiently as possible. ATP-dependent DNA translocases or fork remodeling enzymes, DNA binding proteins, DNA nucleases, and helicases are among the key players called upon to deal with stalled forks. For further reading on the more global topic of fork management, we recommend some recently published reviews on the topic [126,127,128,129,130].

Replication fork stabilization under conditions of stress (e.g., nucleotide deprivation or genotoxin exposure that induces replication-blocking lesions) allows for fork protection from deleterious nucleolytic resection and timely restart of stalled forks [126,127,128]. Besides their canonical ICL repair functions, proteins of the FA pathway play a central role in stabilizing stressed replication forks, thereby ensuring genome integrity. The FA pathway is rapidly activated (as evidenced by FANCD2 mono-ubiquitination and FANCD2 nuclear foci formation) upon cellular exposure to HU or APH that causes replication fork stalling but does not generate ICLs [131,132]. Moreover, increased chromosomal instability observed in FA-deficient cells experiencing replication stress is attributed to impaired stabilization of the stressed replication forks [49,131,133,134]. 

A direct role of the FA/HR pathway in protecting stalled forks entered the spotlight through two breakthrough discoveries from the Jasin lab. Using the single-molecule DNA fiber assay, Schlacher et al. showed that BRCA2 (FANCD1) protects stalled replication forks from MRE11-mediated over-resection by stabilizing Rad51 nucleofilament [48]. A similar fork protective function of FANCD2 and BRCA1 (FANCS) was subsequently described in their second study [49]. Like BRCA2, stalled fork protection by FANCD2 also requires RAD51 filament stabilization and is epistatic with RAD51 [49]. Furthermore, a non-epistatic interaction between FANCD2 and BRCA2 has been delineated [135,136]. In the absence of BRCA2, FANCD2 protects stalled forks from MRE11-dependent degradation [135]. Although blocking MRE11 function completely rescues the fork degradation phenotype in cells deficient of either *BRCA2* or *FANCD2*, only partial recovery of the phenotype is observed in cells lacking both factors [135]. Thus, an additional nuclease(s) other than MRE11 plays a role in fork resection governed by BRCA2 and FANCD2.

Although fork de-protection leads to increased chromosomal instability in FA-defective cells, they are not overtly sensitive to replication stalling agents such as HU, camptothecin, or gemcitabine [49], suggesting redundant pathways ensure cell viability but perhaps at the cost of chromosomal instability. This implies a crucial molecular link between FA pathway loss and tumorigenesis under conditions of replication stress when chromosomal aberrations/mutations arise due to severe fork instability. FANCA loss or a ubiquitination defect in FANCD2 also results in stalled fork degradation upon HU treatment [49]. This suggests that factors functioning upstream of FANCD2 mono-ubiquitination in the FA pathway are also involved in stalled fork protection [49]. Consistent with this notion, the FA core complex protein FANCB was shown to operate in collaboration with RECQL5 helicase to prevent nascent strand degradation upon HU treatment [137]. Very recently a fork protective role of FANCJ has also emerged (see FANCJ section) [138]. Furthermore, a novel protein designated Biorientation Defect 1-like (BODL1), associated with nascent DNA and sharing homology in its N-terminal region with the mitotic regulator BOD1, was discovered [139]. A role of BODL1 in the DNA damage response is suggested by the presence of multiple ATM/ATR phosphorylation sites. It was proposed that BODL1, like FANCJ (another downstream component of the FA/HR pathway) is involved in RAD51-dependent stalled fork protection. In the absence of BODL1, BLM/FBH1-mediated destabilization of RAD51 nucleofilament causes nascent DNA strand degradation behind the stalled fork and promotes genomic instability. However, BODL1, unlike BRCA2 and FANCD2, protects stalled forks from DNA2 nucleolytic degradation instead of MRE11 nuclease attack. Thus, different FA pathway proteins prevent stalled fork degradation by counteracting distinct nucleases and possibly act in concert to provide stability to the stressed forks. Intriguingly, although they counteract the function of two distinct nucleases at stalled fork, BODL1 depletion does not further exacerbate fork degradation in BRCA2-deficient cells [139]. This observation suggests that when both BRCA2 and BODL1 functions are lost, either one of the nucleolytic resection pathways dominates over the other or both pathways act in concert in a controlled manner at stalled forks. In this context, Liao et al. recently proposed that a negative feedback mechanism restrains elevated resection to prevent deleterious single-stranded DNA accumulation under conditions when both fork protection factors are absent [128]. However, further research to identify additional nucleases functioning at stressed replication forks in defined genetic backgrounds is required to obtain a better understanding of the functional interplay among different FA pathway proteins in protecting stalled replication forks.

Besides its role in preventing deleterious nucleolytic resection of stalled forks, proteins of the FA pathway have additional functions to counteract replication stress. FANCD2 cooperates with BLM to facilitate stalled fork restart and suppress new origin firing [140]. Importantly, while FANCD2 prevents stalled fork degradation independently of BLM, BLM promotes fork progression following restart in a FANCD2-independent manner [49,140]. Thus, FANCD2 and BLM have cooperative as well as independent roles in the context of stalled fork recovery. Furthermore, upon replication fork stalling the FA-associated nuclease FAN1 interacts directly with the chromatin-bound FANCD2‒BLM complex and cooperatively promotes replication fork restart [141]. Here, FAN1 chromatin access is tightly regulated by FANCD2, which prevents FAN1 nuclease-mediated over-resection of the stalled fork [141]. In the absence of FANCD2, uncontrolled nucleolytic activity of FAN1 along with MRE11 results in nascent DNA strand degradation behind the fork [141]. Lachaud et al. further showed that ubiquitinated FANCD2 promotes stalled fork processing by directly recruiting FAN1 nuclease to stalled replication forks [134]. This in turn restrains DNA replication under stressed conditions and prevents chromosomal instability [134]. Apart from FAN1, CtIP nuclease is also implicated in the FANCD2-driven recovery of stalled replication forks [142]. Importantly, FANCD2-mediated stalled fork recovery is completely independent of the FA core complex, suggesting the process operates with downstream FA pathway proteins such as BRCA2 and FANCJ [143].

In response to replication stalling, experimental evidence suggests that FA proteins directly interact with the replisome and regulate DNA synthesis. Data obtained from experiments utilizing a technique known as isolation of proteins on nascent DNA (iPOND) followed by mass spectrometry (MS) analysis revealed that in HU-treated cells FANCD2 and FANCI are recruited to newly synthesized DNA in response to nucleotide depletion [144]. Moreover, in response to HU treatment, FANCD2 binds to the MCM2-7 replicative helicase complex and regulates its function to restrain DNA synthesis at stalled forks thereby preventing accumulation of single-stranded DNA gaps and genomic instability [144]. While FANCD2 restricts DNA synthesis, FANCI induces MCM2-7 helicase activity to promote dormant origin firing to help rescue stalled replication forks in response to mild replication stress [145]. Although not mechanistically clear, this function of FANCI in promoting dormant origin firing is inhibited by FANCD2 [145]. However, under conditions of severe stress (e.g., > 2 mM HU) exposure or prolonged (>18 h) HU incubation), activated ATR signaling phosphorylates FANCI to suppress new origin firing and provide cells with enough time to repair the damage and resume replication [145]. DNA synthesis by the translesion DNA polymerase kappa in response to HU-induced fork stalling is regulated by mono-ubiquitinated FANCD2 (as well as PCNA polyubiquitylation), suggesting that FA pathway activation is required for replication stress recovery [146]. Thus, the FA pathway functions at three distinct levels to recover stalled replication forks: i) fork protection from nucleolytic degradation, ii) stalled fork restart, and iii) suppression of new origin firing. Future studies will provide more mechanistic insights into these important non-canonical functions of the FA pathway to deal with replication stress. 

## 4. New Developments in Understanding FANCJ’s Role in the Replication Stress Response

Although much insight to the roles of RecQ helicases in managing fork stress has been garnered over the years [102], up to only very recently have there been advances in the characterization of other auxiliary helicases such as those from the iron‒sulfur (Fe‒S) family, which includes FANCJ, RTEL1, DDX11, and XPD [147]. Among these, FANCJ is implicated in both hereditary disease and cancer and thought to have a specialized role(s) during DNA replication [148]. Since the discovery of FANCJ mutations linked to FA, there have been numerous studies on the molecular and cellular functions of the Fe‒S DNA helicase that address its broader roles in genome metabolism. Beyond its involvement in ICL repair through a still poorly understood series of downstream events to mediate HR repair of the ICL-induced DSB, it is now evident that FANCJ is a key player in the replication stress response. In this section, we will summarize the latest developments on FANCJ and mechanistic aspects of how the helicase is proposed to operate at stalled replication forks. The reader is referred to earlier reviews on FANCJ to provide a historical backdrop for this perspective [149,150,151].

Recently, we addressed the catalytic requirements of FANCJ necessary for its function in DNA repair versus response to replication stress [152]. By characterizing patient-derived FANCJ missense mutations residing in the helicase core domain both biochemically and genetically, we determined that a minimal threshold of FANCJ helicase activity is required for the cellular response to the DNA polymerase inhibitor APH, G-quadruplex ligand telomestatin (TMS), or the DNA strand-breaking agent bleomycin (BLEO). A catalytically inactive FANCJ-H396D mutant failed to rescue FANCJ null cells for any of these agents, whereas a partially active FANCJ-R707C mutant characterized by a dimerization defect and reduced processivity was competent to confer significant cellular resistance to APH, TMS, or BLEO. Interestingly, expression of either FANCJ-H396D or FANCJ-R707C completely failed to restore resistance to the DNA cross-linking agents MMC or cisplatin, indicating that optimal FANCJ helicase activity is required for ICL repair. This observation is consistent with a previous study in which it was shown that a patient-derived FANCJ missense mutation in the Fe-S domain that cripples helicase activity but leaves ATPase and single-stranded DNA translocase functions intact abolishes its ability to restore resistance of FANCJ null cells to an ICL-inducing agent [153]. Furthermore, these findings reinforce the belief that sensitivity of FANCJ patient cells to ICL-inducing agents is an excellent biomarker for FA. Further studies in this area should address if other missense mutations in FANCJ (linked to FA or associated with breast or ovarian cancer) that specifically interfere with key protein interactions of FANCJ but leave the catalytic function intact are able to differentially function in ICL repair versus response to agents that impede but do not entirely arrest cellular DNA replication. In terms of the replication fork, we surmise that it makes sense that the role of FANCJ in fork reconstruction via HR repair of ICL-induced damage would require maximal catalytic DNA unwinding (or possibly branch-migration activity), whereas the role of the helicase to help remodel or protect stalled forks during replication stress would only require partially active FANCJ helicase. New studies from the Cantor lab (discussed below) may begin to shed light on FANCJ’s role at stalled forks.

The Cantor lab took a novel pair-wise approach to address FANCJ’s role in replication [138]. They examined the effects of FANCJ deficiency on fork protection as measured by the popular DNA fiber analysis and determined the identity of unique proteins associated with replication forks by iPOND in CRISPR (Cas9) FANCJ knockout (KO) cells compared to the same cells endogenously expressing wild-type FANCJ. The DNA fiber results showed that nascently synthesized DNA is degraded in FANCJ-KO U2OS cells exposed to 4 mM HU in a MRE11 nuclease-dependent manner. The fork degradation was also observed in FA-J patient fibroblasts; suppression of fork degradation was dependent on expression of helicase-active FANCJ. In terms of the iPOND results, the abundance of several proteins in unstressed FANCJ-deficient cells was decreased, including ones involved in chromatin remodeling (e.g., SMARCAL1) and HR promotion (KIF4A). Notably, the replication fork remodeling enzyme HLTF was enriched at the fork and found to be preferentially associated with chromatin in FANCJ-KO cells. Moreover, DNA fiber analysis showed that HTLF depletion in HU-treated FANCJ-KO cells decreased fork degradation; however, it was not determined if ATP hydrolysis-dependent catalytic function of HLTF was involved. Further study demonstrated that HLTF helps FANCJ-KO cells recover from stress imposed by HU. Finally, Peng et al. provided experimental evidence that the associated unrestrained replication and single-stranded DNA gaps characteristic of HLTF-depleted cells were prevented by FANCJ deficiency. This led the authors to propose a model in which the FANCJ DNA helicase and HLTF fork remodeler keep each other in check to balance fork degradation and DNA synthesis elongation under conditions of replication stress. 

There are several important questions to be addressed regarding the significance of the FANCJ-HLTF fork protection model in vivo and from a biomedical perspective: 1) Does the model apply under conditions of replication stress imposed by non-B-form DNA structures such as endogenous G-quadruplexes (in which FANCJ is already implicated [154,155,156,157,158,159]) or covalent DNA damage imposed endogenously (e.g., formaldehyde by-products [12]) or exogenously by compounds such as those used in chemotherapy [7]? 2) Are chromosomal fragile sites contributing to genomic instability prevalent in cancer and other disease states and aggravated by replication stress found in greater abundance when FANCJ and HLTF activities are not balanced properly? 3) Do defects in the coordinate action of FANCJ and HLTF contribute to FA, and are there other chromosomal instability disorders that by analogy arise due to deficiencies in balancing catalytic activities of specific molecular motors at the stalled replication fork? 4) Do FANCJ and HLTF represent druggable targets to enhance anticancer treatments, as proposed for other DNA damage response proteins [160]. Broadly speaking, an improved understanding of fork protection and its importance for human disease and cancer will help researchers and clinicians to bridge gaps in our knowledge bringing enlightenment for better diagnosis, treatment, and cures.

Finally, while small molecule inhibitors of RecQ helicases WRN [107,161,162] and BLM [163] have attracted attention as an approach to enhance anticancer therapies, we propose that FANCJ may also be a useful target for pharmacological inhibition. Given its importance in ICL repair induced by chemotherapy drugs (e.g., MMC, cisplatin), the emerging role of FANCJ to help cells deal with stalled forks suggests that the helicase may be a good target for inhibition to enhance the killing effects of drugs that perturb fork progression in rapidly dividing cancer cells. Here it may be advantageous to identify FANCJ-specific DNA helicase inhibitors using molecular docking interfaced with a computational approach; however, as we await a solved FANCJ structure, the approach would have to rely on a homology model of the FANCJ helicase core domain based on the sequence-related Fe‒S helicase XPD [164]. 

**Figure 2:**
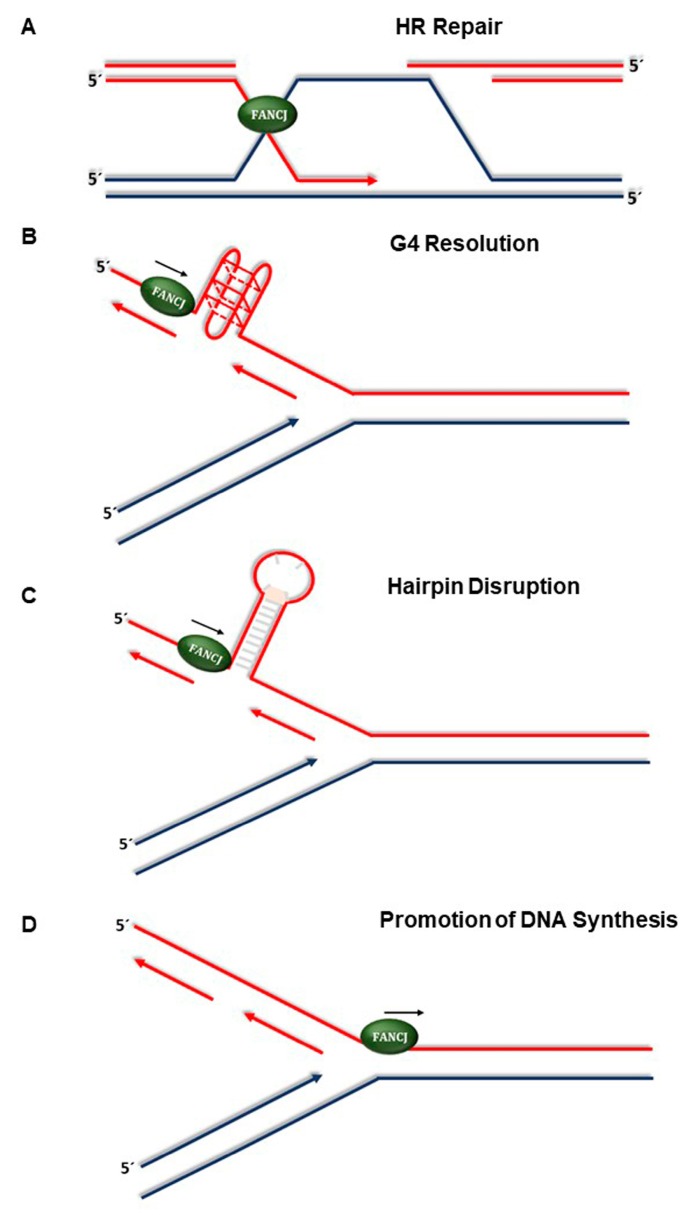
Roles of FANCJ in the replication stress response. (**A**) FANCJ facilitates homology-directed repair of DSBs or ICL-induced broken replication forks. (**B**) FANCJ resolves G-quadruplex structures formed at transient single-stranded DNA regions during replication or possibly transcription. (**C**) FANCJ unwinds hairpin duplexes formed in single-stranded DNA templates during replication fork progression through repetitive DNA sequences of the genome thereby suppressing microsatellite instability. (**D**) FANCJ promotes DNA synthesis at stalled replication forks to minimize HLTF-mediated fork regression and prevent fork degradation.

In addition to its fork protection role, FANCJ is important for fixing broken replication forks via HR repair of DSBs during the S and G2 phases of the cell cycle. This idea builds upon the original observation that FANCJ physically binds to the tumor suppressor BRCA1, which is also implicated in HR repair [80]. Most recently, the Nagaraju lab acquired experimental evidence that FANCJ regulates sister chromatid recombination in a manner that is dependent on its helicase activity and interaction with BRCA1 [165]. Flipping the coin, FANCJ may play a role in preventing fork stalling in the first place by resolving alternate DNA structures such as G-quadruplexes, as mentioned above. Recently, two laboratories independently showed that FANCJ prevents microsatellite instability during replication to suppress chromosome breakage and translocations, presumably by resolving non-B DNA structures in a manner that is independent of the classic FA pathway used to repair ICLs [166,167]. Thus, FANCJ operates at four distinct levels during replication stress or DNA repair to maintain genomic stability (Figure 2).

## 5. Involvement of FA Proteins in the Response to R-Loop-Induced Replication Stress

**Figure 3:**
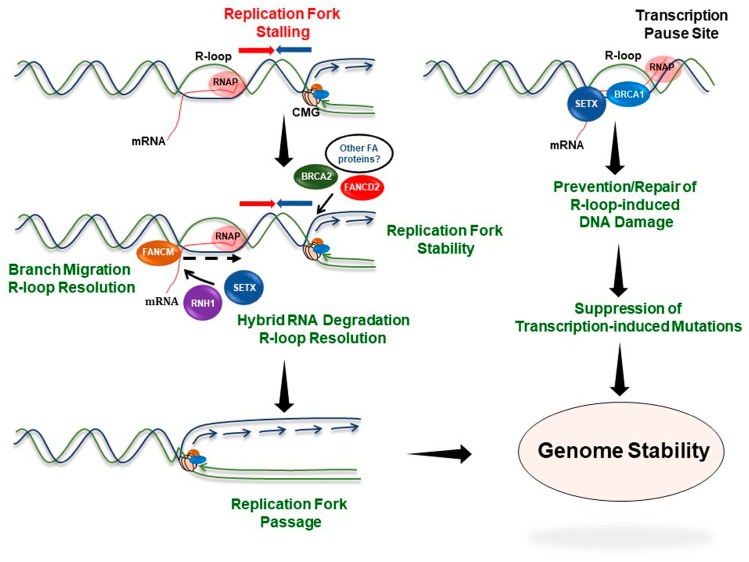
The Fanconi anemia pathway suppresses R-loop-associated genome instability. A replication fork encountering a R-loop is stabilized by the coordinated functions of proteins implicated in FA. BRCA2, FANCD2, and possibly other FA proteins are recruited to the replication fork encountering a DNA‒RNA hybrid to prevent fork collapse. The FA proteins may facilitate R-loop resolving enzymes such as RNH1 or SETX. On the other hand, FANCM directly resolves R-loop structures to facilitate resumption of DNA synthesis. Thus, proteins involved in the FA pathway mitigate R-loop-associated transcription‒replication conflicts and ensure genome stability. BRCA1 in association with SETX is recruited to the R-loop sites formed at transcriptional termination regions of highly transcribed genes and acts to repair/prevent R-loop-driven DNA damage at these genomic loci.

Recent studies highlighted a crucial non-canonical role of the FA pathway in regulating R-loop-driven genomic instability [168,169] (Figure 3). R-loops are transcription-associated three-stranded nucleic acid structures that form when nascent RNA anneals with the template DNA strand (DNA: RNA hybrid) while displacing the other strand of the duplex DNA. Although programmed R-loop formation is beneficial for certain biological processes including control of gene expression, abnormal accumulation of these hybrid structures causes DNA damage and genomic instability [170,171,172]. Moreover, persistent R-loops impair transcription and obstruct replication fork movement, thereby generating replication stress [170,172,173,174,175,176]. However, cells have multiple ways to either prevent the unscheduled formation of the co-transcriptional R-loops or efficiently remove them from the genome to minimize replication‒transcription conflicts [177].

Among these mechanisms, BRCA2 (FANCD1) was the first FA protein implicated in the suppression of R-loop-associated genome instability [178]. Using the procedure of proximity ligation assay (PLA) with a fluorescently tagged hybrid-interacting RNaseH1 peptide and a hybrid-specific antibody, Bhatia et al. showed that BRCA2 functionally interacts with a TREX-2 mRNA export factor PCID2 and prevents R-loop accumulation in vivo. Furthermore, results from DNA-RNA immunoprecipitation (DRIP) experiments revealed hybrid enrichment at actively transcribed genes in *BRCA2*-deficient cells [178]. The authors proposed a model whereby BRCA2 and other FA proteins directly act on the replication fork ahead of a R-loop and thereby stabilize the fork, prevent R-loop extension, promote R-loop dissolution and possibly facilitate fork restart in replicating cells [178]. On non-replicating chromatin, BRCA2 might get recruited or stabilized in the vicinity of transcribed regions by TREX-2 complex, where it promotes R-loop dissolution by providing greater access of R-loop resolving enzymes to the hybrid sites [178]. Like BRCA2, the downstream FA pathway protein BRCA1 (FANCS) is also implicated in R-loop metabolism [179]. Together with DNA/RNA helicase senataxin (SETX), BRCA1 prevents/repairs R-loop-induced DNA damage at transcription pause sites of actively transcribed genes. This is consistent with insertions/deletions found particularly at the hybrid containing transcription termination regions in breast tumors carrying germline *BRCA1* mutations [179]. *BRCA1*/*SETX* knockdown results in a single-stranded DNA break and R-loop accumulation at transcription termination loci. Because recruitment of BRCA1/SETX complex to R-loop sites was shown to be BRCA1-dependent, it is plausible that BRCA1 prevents R-loop accumulation by facilitating hybrid sites access to SETX. It should be noted, however, that BRCA1/SETX depletion also causes DSBs but they are not R-loop-associated.

Schwab et al. further demonstrated that under normal physiological conditions, components of the FA pathway are required to mitigate transcription‒replication conflict and limit unscheduled R-loop accumulation [169]. Results obtained from PLA experiments revealed that FANCD2 colocalizes with elongating RNA polymerase II in vivo in a transcription-dependent manner. Furthermore, inhibition of transcription alleviated replication fork progression defects and replication stress observed in *FANCD2*-deficient cells. These observations suggest that under normal growth conditions, transcription complexes are natural impediments to DNA replication and that the FA pathway plays a critical role to overcome these barriers. Apart from this, experimental evidence suggests that the FA pathway is directly involved in preventing unusual accumulation of co-transcriptional R-loops [169]. Overexpression of the R-loop resolving nuclease RNase H1 suppressed FANCD2 nuclear foci formation and mono-ubiquitination in replicating cells. Moreover, RNase H1 overexpression prevented R-loop accumulation and suppressed replication fork arrest and chromosomal instability in *FANCD2* knockdown cells. Consistent with these results and suggesting a broader role of the FA pathway in R-loop metabolism, FA patient-derived *FANCD2*-/- or *FANCA*-/- cells display elevated hybrid accumulation and associated chromosomal aberrations [168]. 

In addition to suppressing R-loop formation, the FANCM DNA translocase directly resolves DNA:RNA hybrids arising from transcription [169]. FANCM-deficient cells exhibit increased immunofluorescence staining by a R-loop-specific antibody. Moreover, results from in vitro unwinding assays showed that purified FANCM efficiently resolves DNA: RNA hybrid substrates [169], further supported by a very recent report demonstrating that FANCM has a R-loop metabolizing function in vitro and that *FANCM*-deficient cells are highly sensitive to R-loop stabilizing drugs [180]. Apart from R-loop resolution, a functional FA pathway might essentially provide stability to the replication forks stalled at hybrid sites, thereby preventing replication fork collapse and DNA breaks [169]. Importantly, the role of FANCD2 in facilitating replication at common fragile sites (CFS) is also attributed to its R-loop removal function [133]; however, the precise role of FANCD2 is not well understood. Overall, the experimental data suggest that R-loop-associated replication‒transcription conflicts that persist when the FA pathway is defective contributes to the elevated chromosomal instability observed in cells from FA patients. 

R-loop-induced genomic instability is implicated in various human diseases including cancer [181]. Given the role of cancer predisposition factors such as BRCA1/BRCA2 and FA pathway in mitigating R-loops, it is reasonable that persistent R-loops are major contributing factors to cancer-associated genomic instability. Indeed, as mentioned above *BRCA1*-deficient breast tumors are highly enriched for mutations at R-loop sites located at transcriptional termination regions in the genome [179]. This suggests that unresolved R-loops might be the chief source of mutagenesis in cancer when tumor suppressors like *BRCA1* are lost. Additional whole-genome sequencing of human cancers harboring driver mutations in major cancer susceptibility genes would be helpful to further dissect the molecular link between R-loop biology and cancer-associated genome instability. Oncogene-induced replication stress is considered as an early driver of genomic instability in cancer [182]. In this scenario, global upregulation of transcription activity and increased R-loop accumulation have been shown to be major driving factors of replication stress upon oncogene activation [183]. Thus, suppressing unscheduled R-loop formation by crucial cellular pathways such as FA under conditions of stress is vital to prevent neoplastic transformation. Better understanding of the molecular relationships between the FA pathway and R-loop biology in the context of FA pathogenesis and cancer will be an exciting field of research.

## 6. Conclusions

Although FA is a complex genetic disorder with many genes involved and an array of cellular phenotypes and clinical symptoms, the underlying cause at the cellular level seems to revolve around molecular defects contributing to a compromised DNA damage response and DNA repair. While evidence suggests that ICLs are the major culprit, there are other drivers of replication stress and genomic instability in FA. In this review, we have discussed the roles of various FA gene products and associated DNA repair factors in the metabolism of not only ICLs but also other bulky lesions, unusual replication fork structures caused by replisome-stalling agents, and R-loops. Continued research in this area will help to elucidate potential strategies to ameliorate the molecular and cellular defects of FA. Furthermore, fundamental advances in understanding the basis for the chromosomal instability leading to various cancers associated with mutations in genes (particularly downstream members) of the FA pathway and their interacting partners will help to identify new biomarkers and therapeutic strategies.
